# Precision and reproducibility of semi-automated late gadolinium enhancement quantification techniques in patients with hypertrophic cardiomyopathy

**DOI:** 10.1186/1532-429X-16-S1-P340

**Published:** 2014-01-16

**Authors:** Yoko Mikami, Louis Kolman, Sebastien X Joncas, Carmen Lydell, Sarah Weeks, James A White

**Affiliations:** 1 Stephenson CMR centre, Libin Cardiovascular Institute of Alberta, Calgary, Alberta, Canada; 2 Cardiac Sciences, University of Calgary, Calgary, Alberta, Canada; 3 Diagnostic Imaging, University of Calgary, Calgary, Alberta, Canada

## Background

The presence and extent of hyper-enhancement (HE) on Late Gadolinium Enhancement (LGE) has been associated with adverse events in patients with Hypertrophic Cardiomyopathy (HCM). Signal-threshold techniques are routinely employed for quantification; however, the precision and reproducibility of these versus a gold standard remains uncertain. Full Width Half of Maximum (FWHM) techniques are suggested to provide greater reproducibility than Signal Threshold versus Reference Mean (STRM) techniques, however the precision of these approaches versus manual assignment of optimal signal intensity(SI) thresholds has not been studied. We compare all known semi-automated LGE-quantification techniques for precision and reproducibility among patients with HCM.

## Methods

Seventy-six patients with HCM (51 male, age 54 ± 13) were studied. Endocardial and epidcardial borders were manually traced. Total HE volume was quantified using 6 semi-automated techniques and compared to expert manual adjustment of the signal intensity threshold. Techniques tested included STRM-based thresholds of > 2, 3, 5 and 6 SD above mean SI of reference myocardium, the FWHM technique, and the Otsu-auto-threshold (OAT) technique. The SI threshold applied for each slice and total HE volume were recorded. Bland-Altman analysis and intra-class correlation coefficients (ICC) were reported for each semi-automated technique versus expert, manually adjusted HE segmentation. Intra- and inter-observer reproducibility assessments were performed.

## Results

**Figure 1:**
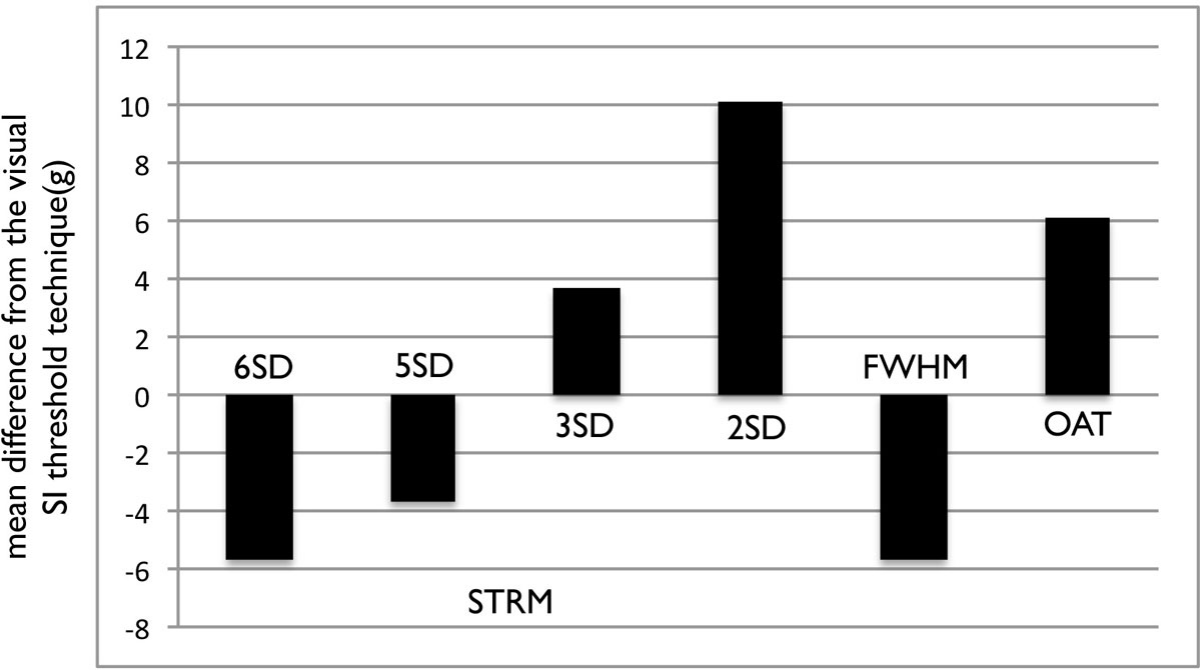
**The mean difference of the total enhanced mass between each semi-automated technique and manual segmentation**.

Fifty-two patients showed HE on a total of 201 slices. Total HE volumes by manual segmentation were reproducible (mean difference between observers = 0.06 g, within observer = 1.35 g). For precision, the STRM > 3SD technique showed the greatest agreement with the manual segmentation (ICC = 0.95, mean difference and 95% limits of agreement = 3.7 ± 12.1 g) while STRM > 6SD, > 5SD and FWHM techniques systematically underestimated total HE volume (Figure 1). STRM > 2SD and OAT techniques over-estimated HE volume. Slice-based analysis similarly showed the STRM > 3SD threshold to most closely approximate manually adjusted SI thresholds (ICC = 0.91). For reproducibility, the FWHM method showed the greatest intra- and inter-observer reproducibility for total HE volume (mean difference = 0.3 g and 0.7 g, respectively). Reproducibility values for STRM-based thresholds improved with increasing SI threshold. The intra- and inter-observer reproducibility of the 3SD threshold demonstrated an acceptable mean difference of 1.14 g and 1.14 g, respectively.

## Conclusions

FWHM segmentation provides superior reproducibility, however systematically under-estimates total HE volume compared to manual segmentation in patients with HCM. The STRM > 3SD technique provides the greatest precision while retaining acceptable reproducibility and may therefore be the preferred approach for HE quantification in this population.

## Funding

Canada Foundation for Innovation.

**Figure 2:**
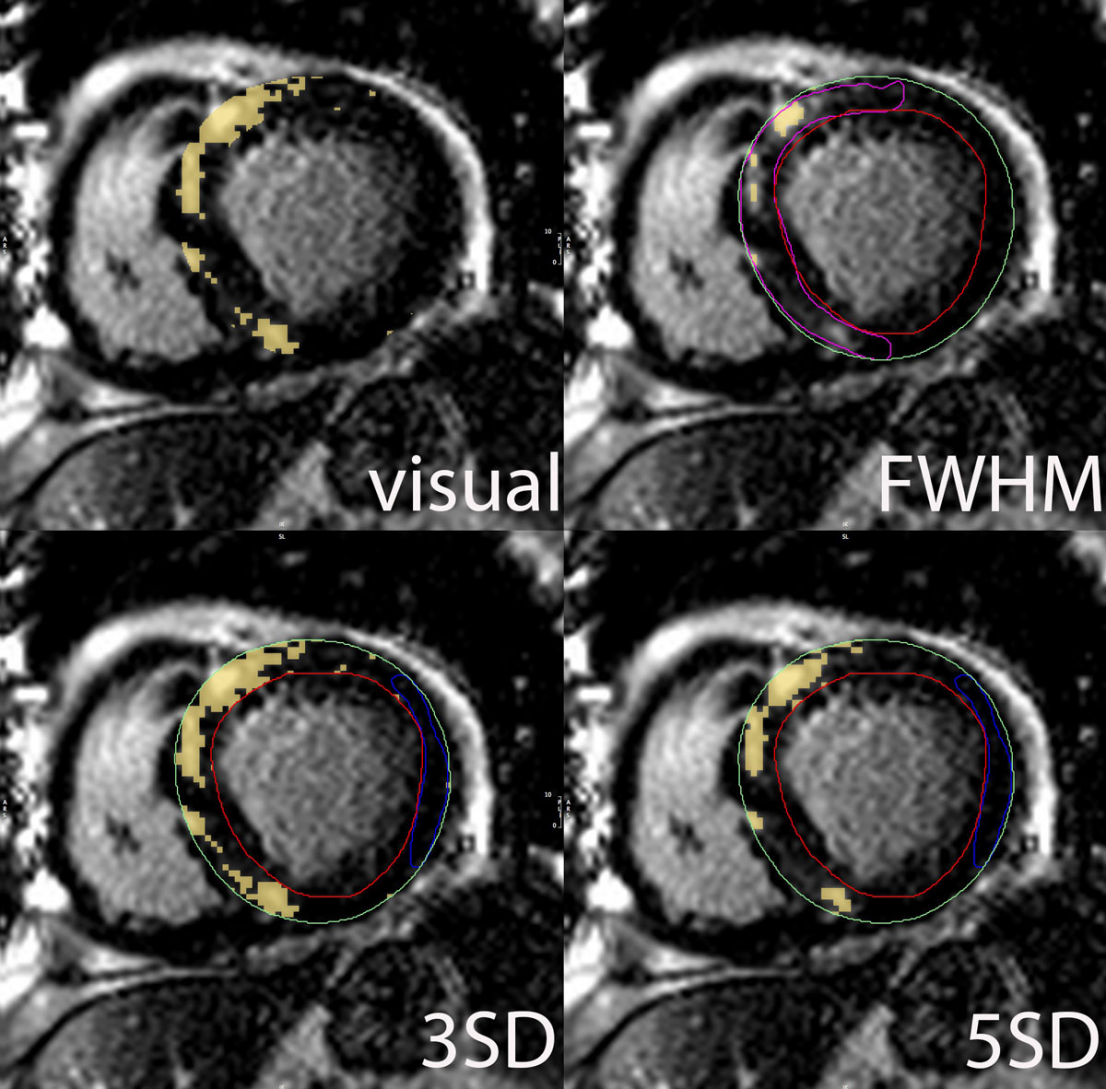
**Manual segmentation and semi-automated quantification of hyper-enhancement**. STRM > 3SD technique shows the greatest agreement with the manual segmentation.

